# Testing the Process Dissociation Procedure by Behavioral and Neuroimaging Data: The Establishment of the Mutually Exclusive Theory and the Improved PDP

**DOI:** 10.3389/fpsyg.2020.474538

**Published:** 2020-11-26

**Authors:** Jianxin Zhang, Xiangpeng Wang, Jianping Huang, Antao Chen, Dianzhi Liu

**Affiliations:** ^1^School of Education, Jiangnan University, Wuxi, China; ^2^School of Linguistic Sciences and Arts, Jiangsu Normal University, Xuzhou, China; ^3^School of Education, Soochow University, Suzhou, China; ^4^Key Laboratory of Cognition and Personality of Ministry of Education, Faculty of Psychology, Southwest University, Chongqing, China

**Keywords:** the classical PDP, the mutually exclusive theory, the improved PDP, four knowledge types, ALFFs in the resting-state

## Abstract

The process dissociation procedure (PDP) of implicit sequence learning states that the correct inclusion-task response contains the incorrect exclusion-task response. However, there has been no research to test the hypothesis. The current study used a single variable (Stimulus Onset Asynchrony SOA: 850 ms vs. 1350 ms) between-subjects design, with pre-task resting-state fMRI, to test and improve the classical PDP to the mutually exclusive theory (MET). (1) Behavioral data and neuroimaging data demonstrated that the classical PDP has not been validated. In the SOA = 850 ms group, the correct inclusion-task response was at chance, but the incorrect exclusion-task response occurred greater than chance. In the SOA = 850 ms group, the two responses were not correlated, but in the SOA = 1,350 ms group and putting the two groups together, the two responses were in contrast to each other. In each group, brain areas whose amplitude of low frequency fluctuations (ALFFs) in the resting-state related to the two responses were either completely different or opposite to one another. However, the results were perfectly consistent with the MET proposed by the present study which suggests that the correct inclusion-task response is equal to the correct exclusion-task response is equal to *C* + *A*_1_, and the incorrect exclusion-task response is equal to *A*_2_. *C* denotes the controlled response and *A*_1_ and *A*_2_ denote two different automatic responses. (2) The improved PDP was proposed to categorize the 12 kinds of triplets as delineating four knowledge types, namely non-acquisition of knowledge, uncontrollable knowledge, half-controllable knowledge, and controllable knowledge with the MET. ALFFs in the resting-state could predict the four knowledge types of the improved PDP among two groups. The participants’ control of the four knowledge types (degree of consciousness) gradually improved. Correspondingly, the brain areas in the resting-state positively related to the four knowledge types, gradually changed from the sensory and motor network to the somatic sensorimotor network, and then to the implicit learning network, and then to the consciousness network. The brain areas in the resting-state negatively related to the four knowledge types gradually changed from the consciousness network to the sensory and motor network. As SOA increased, the brain areas associated with almost all the four knowledge types changed. (3) The inhomogeneous hypothesis of the MET is best suited to interpret behavioral and neuroimaging data; it states that the same components among the four knowledge types are not homogeneous, and the same knowledge types are not homogeneous between the two SOA groups.

## Introduction

### The Process Dissociation Procedure (PDP) in Implicit Sequence Learning

Consciousness is fundamental to an individual’s survival, learning and development. When unconscious knowledge advances to conscious knowledge, it can be flexibly controlled, integrated, and transferred (Dehaene et al., 2011, 2014). Implicit sequence learning is an important paradigm to reveal the mechanisms of consciousness emergence and change (Fu and Fu, 2006; Voss and Paller, 2009; Zhang et al., 2016). It involves a sequence rule in a certain dimension of stimulus such as its location, but the participants do not know the sequence rule and they are just asked to respond to the location of the stimulus; therefore, they perform an implicit sequence learning rather than an explicit sequence learning. The common used sequence is the SOC sequence such as 3-4-2-3-1-2-1-4-3-2-4-1 (Reed and Johnson, 1994), whereby each location is completely determined by the previous two locations, so three consecutive locations constitute a smallest-rule unit, namely a triplet. As the implicit sequence learning proceeds, different participants can generate knowledge at different levels of consciousness for the sequence (Norman et al., 2006; Fu et al., 2013; Zhang et al., 2014), and a participant can also generate knowledge at different levels of consciousness for different parts of the sequence (Zhang, 2015). Therefore how to define and measure the levels of consciousness is the premise of consciousness research in implicit sequence learning.

A classic method to measure consciousness in implicit sequence learning is the process dissociation procedure (PDP) obeying oppositional logic (Jacoby et al., 1989; Jacoby, 1991). Oppositional logic defines consciousness as a controlled response (shorthand for *C*) which can be regulated by explicit policies, and unconsciousness as an automatic response (shorthand for *A*) which cannot be regulated by explicit policies. They work against each other in exclusion task, but work together in inclusion task (see below). Destrebecqz and Cleeremans (2001, 2003) introduced PDP into implicit sequence learning consciousness research for the first time and created a free-generation task. Wilkinson and Shanks (2004) changed the free-generation task to a trial-by-trial generation task, and Fu et al. (2010) improved it, as follows: after implicit SOC sequence learning, the participants were presented with two trials of sequence fragments, and were asked to respond to the target as quickly and accurately as in the learning phase. Then, four boxes with a question mark inside each were shown to them. In the exclusion task, participants needed to select a location that did not conform to the sequence rules. If participants wrongly select a location that did conform to the sequence rules, this incorrect exclusion-task response constitute the automatic response (*A*) driven by unconscious familiarity. On the contrary, in the inclusion task, the participants were asked to choose the location that conformed to the sequence rules. They could use the controlled response (*C*) by conscious extraction and the automatic response (*A*) by unconscious familiarity at the same time. Therefore, the incorrect exclusion-task response (*A*) is contained within the correct inclusion-task response (*C* + *A*). The PDP provides continuous scale indicators to quantify consciousness in implicit sequence learning; accordingly, it is widely used (Destrebecqz et al., 2005; Norman et al., 2006; Fu et al., 2010, 2013; Yonelinas and Jacoby, 2012; Zhang et al., 2014).

### Questions About the Validity of the PDP in the Current Study

However, we thought that the PDP is not established. First, we explored this topic from the perspective of logic and operations of the inclusion/exclusion task. On the one hand, the PDP states that, because the exclusion task requires participants to choose locations that do not conform to rules, the controlled response helps to avoid choosing locations that conform to the rules, while the automatic response remains in conflict with the controlled response to facilitate choosing locations that conform to the rules. One the other hand, in the inclusion task, the PDP suggests that the automatic and controlled responses synergistically lead to correctly choosing locations that conform to rules (Jacoby, 1991). This logic is apparently contradictory with its operations of inclusion and exclusion tasks. In the exclusion task, in order to choose locations that do not conform to rules, participants must first determine locations that conform to rules to avoid choosing them; so they must first incorporate both automatic and controlled responses to determine locations that obey rules (Fu et al., 2010, 2013). Obviously, cognitive activities by which participants determine locations that obey rules in exclusion tasks are identical to those in inclusion tasks. That is, the correct exclusion-task response and the correct inclusion-task response are basically the same. The incorrect exclusion-task response should completely differ from both the correct exclusion-task response and the correct inclusion-task response. Thus, the PDP is not validated by logical analysis, which considers that the incorrect exclusion-task response (*A*) is contained by the correct inclusion-task response (*C* + *A*). A more logical relationship would be that the correct inclusion-task response is equivalent to the correct exclusion-task response, which is equivalent to *C* + *A*_1_, and the incorrect exclusion-task response is equivalent to *A*_2_; that is, the correct inclusion-task response and the incorrect exclusion-task response are mutually exclusive, and there is no intersection between them. We call it the mutually exclusive theory (MET).

Second, we explored this topic from the perspective of knowledge. Horga and Maia (2012) and Kiefer (2012) reviewed implicit cognitive control studies and suggested that implicit knowledge may be either automatic or controlled. Fu et al. (2010) asked participants to report subjective knowledge types such as guess, intuition, rule or memory (Dienes and Scott, 2005) in an inclusion/exclusion task, and found that there exists automatic implicit knowledge, such as a guess, whose structure (sequence rules) and judgment knowledge (selecting rule locations) are unconscious. By contrast, there exists controlled implicit knowledge, such as intuition, whose structure knowledge is unconscious, but judgment knowledge is conscious. That is, implicit knowledge is not characterized only by the automatic response. The PDP hypothesizes only one implicit knowledge type such as the automatic response; this hypothesis is inconsistent with the abovementioned studies. However, in the MET, *A*_2_ is automatic implicit knowledge against the exclusion task requirement, and *A*_1_ is controlled implicit knowledge meeting the inclusion task requirement, which is consistent with the above mentioned studies.

### Neuroimaging Studies About Consciousness and the PDP in Sequence Learning and Their Shortcomings

To date, not only is there a lack of studies providing experimental evidence to validate the PDP, but there is also a dearth of neuroimaging studies that test it. However, there is a wealth of neuroimaging literatures regarding implicit sequence learning and its consciousness.

Penhune and Steele (2012) found that the cerebellum is responsible for motor formatting optimization, motor control, and error correction; the primary motor cortex (M1) is responsible for storing sequence knowledge; and the striatum is responsible for stimulus-response connection learning and location prediction. Hardwick et al. (2013) conducted a meta-analysis of implicit motor-sequence learning studies, and found that motor regions (premotor area, primary motor cortex, and cerebellum), primary somatosensory regions, and the striatum were activated during such learning.

Almost all neuroimaging studies of consciousness in sequence learning have used the following dichotomy: they defined explicit sequence learning as a conscious activity and implicit sequence learning as an unconscious activity. The regions of the brain which are involved in consciousness were found by comparing the brain areas active during explicit and implicit sequence learning. Such studies found that conscious processing occurs in the medial temporal lobe, including hippocampus (Reber and Squire, 1994; Gagnon et al., 2004; Squire, 2009; Wilkinson et al., 2009), prefrontal lobe (Rose et al., 2005; Carter et al., 2006; Guo et al., 2008; Rünger and Frensch, 2008; Chu and Liu, 2010), and insula (Yang and Li, 2012). However, this dichotomy did now allow the distinction between learning processes and consciousness, and more importantly, there was a failure to recognize that implicit sequence learning could produce many knowledge types, including consciousness. For example, Rose et al. (2010) and Wessel et al. (2012) showed that when unconscious knowledge became conscious in implicit sequence learning, there existed functional connectivity between the right ventrolateral prefrontal cortex and ventral striatum. However, these studies did not examine whole brain, and did not use the PDP. Thus, the dichotomy could not generate a continuous behavior or neuroimaging scale to quantify consciousness of implicit sequence learning (Zhang et al., 2015).

Although the PDP provides a continuous behavior scale to quantify consciousness of implicit sequence learning, to date only two studies have used this method with neuroimaging techniques to assess inclusion and exclusion tasks, after participants completed implicit sequence learning. Destrebecqz et al. (2005) considered the incorrect exclusion-task response as the automatic response, and the correct exclusion-task response as the controlled response. They for the first time found that striatum activity was positively related to the incorrect exclusion-task response, and anterior cingulate/central prefrontal cortex (ACC/MPFC) activity was positively related to the correct exclusion-task response. They further examined consciousness differences between RSI (response-stimulus interval) = 0 ms and RSI = 250 ms. In each group, there were more correct inclusion-task response than incorrect exclusion-task response, which indicated the presence of controlled response. However, at RSI = 0 ms, the incorrect exclusion-task response occurred greater than chance, which indicated that the automatic response occurred, whereas at RSI = 250 ms, there was no such automatic response. Consistent with the behavioral results, only in the exclusion task at RSI = 250 ms was there functional connectivity between the ACC/MPFC and striatum. That is, the ACC/MPFC controlled activity of the striatum (responsible for sequence knowledge), and the ACC/MPFC were related to consciousness. However, the study did not examine the whole brain, nor did it identify brain areas associated with inclusion tasks. Because of this omission, it could not test the relationship between inclusion and exclusion tasks, nor test the PDP via brain imaging.

With task-state fMRI, Huang et al. (2017) found that, for the 850 ms SOA group, beta-value difference of left medial frontal gyrus (training phase 2 minus phase 1) positively correlated to the correct inclusion-task response. For the 1,350 ms SOA group in training phase 2, beta values of the left inferior parietal lobule and the left middle frontal gyrus positively correlated with the correct inclusion-task response, but beta values of the right inferior parietal lobule negatively correlated with the incorrect exclusion-task response. For the 1,350 ms SOA group in training phase 3, beta values of left lingual gyrus and left inferior frontal gyrus negatively correlated to the incorrect exclusion-task response. In fact, these results showed that brain areas related to the correct inclusion-task response and the incorrect exclusion-task response were completely different. However, the study did not point this out, and only used PDP to define consciousness as the correct inclusion-task response minus the incorrect exclusion-task response. Because of this omission, it did not test the PDP via neuroimaging.

A method suitable for studying consciousness is resting-state fMRI, which investigates spontaneous activity or functional connections within the brain at rest. If a certain cognitive task is associated with activity in certain brain areas in the resting-state, then these brain areas are associated with the cognitive task. If brain areas whose activity in the resting-state related to two cognitive tasks differ, the brain mechanisms of these two cognitive tasks are different. The rationale is as follows: in the resting-state, participants do not perform any cognitive task, so the spontaneous activity of a brain area is its baseline activity and its functional strength index. If it is related to a cognitive task, it indicates that the brain area is related to the cognitive task; this is to say that if the density and the number of neurons of a brain area are related to a cognitive task, the brain area is related to the cognitive task. The rationale is generally accepted and used by many researchers (Heuvel and Pol, 2010; Li et al., 2016; Liu et al., 2017; Jiang et al., 2018), and the resting-state brain areas related to a cognitive task are usually shown to be its task-state brain areas using task-state fMRI. This means that when the participants perform the cognitive task, its related resting-state brain areas are usually activated, as is also seen in the present study. Therefore, brain spontaneous activity in the resting-state is a stable index to measure the individual cognitive characteristics (Liu et al., 2017). One of the classic indexes is ALFFs (the Amplitude of Low Frequency Fluctuations, 0.01 ∼ 0.1 HZ), including most of the psychological cognitive processes. The higher and lower amplitudes are background noises, including physiological activity.

Resting-state studies of sleep and disturbed consciousness have confirmed that there is a global network for consciousness named the “rich club,” including the dorsolateral prefrontal cortex, inferior parietal lobe, middle temporal lobe, precuneus, insula, thalamus, and brainstem (van den Heuvel and Sporns, 2011; Schröter et al., 2012; van den Heuvel et al., 2012; Dehaene et al., 2014). However, there is no resting-state research in implicit sequence learning consciousness. One related dichotomy study is that of Sami et al. (2014), who used pre- and post-task changes to study the differences between implicit and explicit sequence learning. Functional connections between the caudate nucleus and cingulate cortex were enhanced following implicit sequence learning in the post-task resting-state compared to pre-task resting-state, but functional connections among attention and cognitive control networks were enhanced in explicit sequence learning. Because pre- and post-task resting states changes contained two components, namely learning and consciousness, and the study did not assess consciousness, it could not separate learning from consciousness.

### Improvements Made in the Current Study

To summarize, the PDP has not yet been tested via behavioral or neuroimaging data. It was found that the PDP is not established after our analysis of logic, operations of the inclusion/exclusion task and implicit knowledge type. The MET proposed by the current study considered that a more logical relationship would be that the correct inclusion-task response does not contain the incorrect exclusion-task response in either behavioral or neuroimaging data, that is, the two responses are either independent or in opposition to each other. The correct inclusion-task response is equivalent to the correct exclusion-task response, which is equal to *C* + *A*_1_, and the incorrect exclusion-task response is equal to *A*_2_.

How might we test our reasoning? Because the PDP assumes that the incorrect exclusion-task response is contained by the correct inclusion-task response, we can infer that the former must occur as or less frequently than the latter in behavioral data. Further, brain areas related to the incorrect exclusion-task response must, in part, overlap with brain areas related to the correct inclusion-task response in neuroimaging data. If either of these inferences are not supported, this would provide evidence against the PDP. The response-stimulus interval (RSI) affects sequence knowledge representation and consciousness (Destrebecqz and Cleeremans, 2001, 2003; Destrebecqz et al., 2005; Norman et al., 2006; Fu et al., 2010; Zhang et al., 2014), according to Cleeremans and Jiménez (2002) representation quality theory; hence, two stimulus onset asynchrony SOAs (similar to RSI) were set up to test the PDP in different situations.

The current study used the PDP to test its validity. In the case that the experiments did not support PDP, it was indicated that its calculation for types of knowledge is flawed, that is, the correct inclusion-task response might not be equal to *C* + *A*, or the incorrect exclusion-task response might not be equal to *A*, or the former one did not contain the latter one; however, this did not indicate that the inclusion and exclusion tasks have no meaning, but indicated the cognitive components of the two tasks need new calculations. Thus, this study aimed at improving the classical PDP through new calculation in accordance with the MET.

## Materials and Methods

### Participants

Fifty college students participated in the experiment. All participants were right-handed, with normal or corrected-to-normal vision, normal color perception, normal physical and mental health, were not taking psychotropic drugs, and had never previously participated in any implicit-learning experiment. All participants met the criteria for functional magnetic resonance imaging (fMRI) scanning, namely that they had no metal implants, were not claustrophobic, and had a head size compatible with the head coil. The participants volunteered to take part in the experiment. Each participant completed an informed consent form before the experiment and received a 100-yuan compensation after the experiment. The experiments were in accordance with the ethical guidelines of the Declaration of Helsinki and were approved by the Scientific Review Committee of Faculty of Psychology, Southwest University, China.

The data of two participants in each group were removed. Three participants of these had an accuracy of less than 90% in the implicit sequence learning phase (Weiermann et al., 2010), and one participant had head motion greater than 2 mm in the pre-task resting state. Finally, 46 participants’ data were effective (SOA = 850 ms group: *n* = 23, 8 male, 15 female, age *M* ± *SD* = 21.30 ± 1.96; SOA = 1,350 ms group: *n* = 23, 9 male, 14 female, age *M* ± *SD* = 20.91 ± 1.56). Age difference between the two groups was not significant, *p* = 0.459 > 0.05. There was no significant difference in Big Five personality dimensions between the two groups (for N, E, O, A, C, *p*s = 0.159, 0.572, 0.727, 0.062, 0.672 > 0.05).

### Materials

The materials used in implicit sequence learning were four blue circles (4.6 cm in diameter), which were arranged horizontally. The centers of adjacent circles were 6.9 cm apart, and the two circles on the left were symmetrical to the two circles on the right, relative to the center of the screen. Only the target circle was filled with blue color; the other circles were outlined in blue, with unfilled centers. The target-circle location order followed the SOC sequence rule: 3-4-2-3-1-2-1-4-3-2-4-1 (Reed and Johnson, 1994), in which each target location was determined by two prior target locations, and three target locations formed a smallest-rule unit, namely a triplet.

### Design and Procedure

**FIGURE 1:**
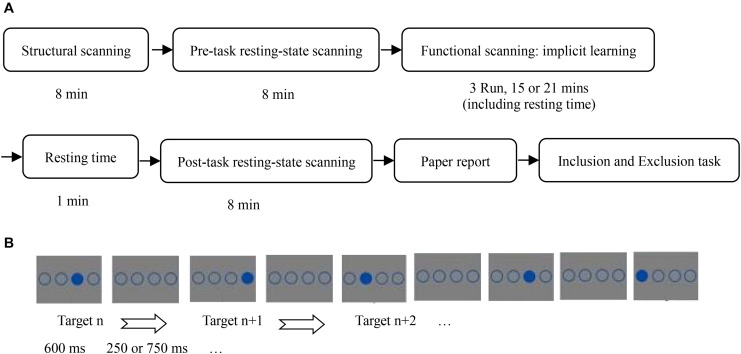
**(A)** Flow chart of all experiments; **(B)** stimulus presentation of implicit sequence learning.

This study used a single variable (SOA: 850 vs. 1350 ms), between-subjects design. The experimental procedure is shown in Figure 1.

When the participants were placed in the fMRI scanner, they were asked to place their hands beside their body, and to put the middle finger and index finger of their left hand on the left mouse buttons 1 and 2, and the index finger and middle finger of their right hand on the right mouse buttons 3 and 4, corresponding to the horizontal positions of the stimulus circles.

In the implicit sequence learning phase, participants completed implicit sequence learning while receiving task-state scanning. The task-state data were discussed in another article (Huang et al., 2017). The instructions stated that the experiment measured response speed and accuracy of pressing a target-location button. The task was to immediately press the button corresponding to the position of the solid circle as quickly and accurately as possible. The participants were told to strictly follow the instructions, otherwise they would not receive payment. In the practice stage, there were 24 trials that obeyed the SOC rule. A random sequence was not used to avoid its influence on implicit sequence learning as a novel stimulus. Further, there were 15 blocks of implicit sequence learning. Each block was composed of 48 trials with a different target-location in the first trial to avoid participants easily noticing the sequence rules. The SOC rule cycled 4 times in each block. Before the start of each block, a fixation cross was presented for 13.2 s as a baseline. Five blocks formed an fMRI scanner run. Before each run, a fixation cross was shown for 7.5 s, to allow the scanner to stabilize. There was a 40 s rest period between runs. In the SOA = 850 ms condition, a solid circle and three unfilled circles were presented for 600 ms, followed by four unfilled circles for 250 ms. Participants were required to make their selection within 850 ms. In the SOA = 1,350 ms condition, a solid circle and three unfilled circles were also shown for 600 ms, followed by four unfilled circles for 750 ms; participants were required to make their selection within 1,350 ms.

After the learning phase, participants sat in front of a computer, with their eyes 70 cm from the center of the screen. A custom experimental program ran under E-prime 2.0 on a PC (Lenovo LX-GJ556D), with a 17-inch color display (resolution 1,024 × 768, refresh rate 60 Hz). The consciousness-assessment stage included a written-report task, an inclusion task, and an exclusion task. In the written-report task, the participants were asked to write down on a piece of paper all thoughts they had while they were taking part in the experiment. The inclusion/exclusion task was the same as that in Fu et al. (2010, 2013). Additionally, in the third element of each triplet, when four boxes with a question mark inside each were shown, participants could not choose the same location as the second element. Therefore, there were only three locations they could choose, and the chance performance level was one-third. The inclusion/exclusion tasks consisted of 72 triplets of the sequence, such that the full set of 12 kinds of triplets of the SOC rule were repeated 6 times.

### Resting-State Data Collection and Analysis

The participants adopted an eyes-closed resting-state. Because no prior resting-state research has considered implicit sequence learning consciousness, the whole brain was examined, i.e., we did not limit consideration to consciousness-related areas found in extant studies. Stimulus onset asynchrony SOA (similar to RSI) was used to fit nuclear magnetic scans.

The fMRI data were collected using a Siemens 3.0 T magnetic resonance imaging scanner and an 8-channel phased front head coil. Pre/post-task resting-state imaging used a gradient echo (GRE) single-excitation echo-planar imaging (EPI). The scan parameters were as follows: TR = 2,000 ms, TE = 30 ms, FA = 90°, FOV = 220 mm × 220 mm, matrix size = 64 mm × 64 mm, depth = 3 mm, planar resolution = 3.13 mm × 3.13 mm, interval scanning, 33 layers, layer spacing = 0.6 mm, total 240 layers. Structural imaging used a 3D TlWI (MP-RAGE) sequence with sagittal scans. Scan parameters were the following: TR = 2,600 ms, TE = 3.02 ms, FA = 8°, no interval, FOV = 256 mm × 256 mm, matrix size = 256 mm × 256 mm, total 176 layers.

Pretreatment and analysis of resting-state data used DPARSF 3.0 Advanced Edition Calculate (Yan et al., 2016) in Original Space (Warp by DARTEL), following standard procedures: First, conversion of raw DICOM-format data to NIFTI format. To allow for signal stabilization of the image, the first 10 TR images were removed, after which time layer correction (slice timing) and head movement correction (realign) were conducted. If head movement greater than 2 mm occurred during resting-state, the data were deleted. Second, the new segment and DARTEL was used to split the structural T1 data without standardization, and register the T1 split data directly to the resting-state functional images. Before registration of structural and functional data, the AC-PC line of each participant’s T1 image and the resting-state function was registered, and then automatic registration was applied. Thus, the resting-state analysis took place in the original T1 space. Third, we adjusted for head motion (adopting Friston 24), linear drift, white matter, and cerebrospinal fluid via regression. Fourth, low frequency fluctuations ALFFs (filter range: 0.01–0.1 Hz) were calculated. Fifth, the resting-state function was registered to the standard MNI space (normalization), using a 3 mm × 3 mm × 3 mm voxel size, with 4 mm × 4 mm × 4 mm full width at half maximum (FWHM) smoothing.

REST1.8 (Song et al., 2011) was first used to extract ALFFs during pre-task resting state in 116 Anatomical Automatic Labeling (AAL) brain areas. For brevity, the pre-task resting-state was called resting-state, the same as follows. Then, SPSS19.0 was used to implement Pearson correlation analyses between ALFFs in 116 AAL brain areas and the correct inclusion-task response/the incorrect exclusion-task response. Finally, SPSS19.0 was used to generate Pearson correlation analyses between ALFFs in 116 AAL brain areas and the improved PDP. Since the original ALFF for each AAL brain area (the average/total ALFF of its all voxels) was extracted (Wang, 2009; Li, 2015; Tang et al., 2018), multiple comparisons correction was not required for the correlation analyses above.

### Data Analysis Steps

There were two data analysis steps: (1) the classical PDP was tested by behavioral and neuroimaging data; (2) if the results were inconsistent with the classical PDP, the improved PDP was necessarily established in accordance with the MET, and its related brain activity was detected.

## Results

### Testing the Classical PDP by Behavioral and Neuroimaging Data

#### Behavioral Results of the Classical PDP Analysis for Generation Tasks

To investigate consciousness in pure implicit sequence learning, none of the novel stimuli, such as improbable sequences or transfer blocks, were presented in the learning phase (Rünger and Frensch, 2008; Rünger, 2012; Huang et al., 2015). Therefore, there was no reaction-time difference between probable and improbable sequences to analyze the extent of learning. Because of practice and fatigue effects, reaction-time difference between block 1 and 15 could not serve as a measure of learning. Instead, we used indexes in the generation tasks to estimate learning.

Because both the correct inclusion-task response and the incorrect exclusion-task response are measures of the learning degree, the greater of the two was used to understand the extent of learning (see Table 1). For example, if the number of the correct inclusion-task response of one participant was greater than or equal to the number of the incorrect exclusion-task response, the former was used as his/her extent of learning; otherwise, the latter was used.

**TABLE 1:** Indexes of generation tasks (*M* ± *SD*).

	SOA = 850 ms (*n* = 23)	SOA = 1,350 ms (*n* = 23)
The correct inclusion-task response	29.00 ± 12.84	33.70 ± 11.35
The incorrect exclusion-task response	28.83 ± 7.11	25.52 ± 10.14
Extent of learning	35.13 ± 4.34	36.43 ± 8.32

There was no difference for the two responses and the extent of learning between the two groups by one-way ANOVA (*p*s = 0.196, 0.207, 0.508 > 0.05). Chance performance for the inclusion/exclusion tasks were one-third. There were 72 triplets in the inclusion/exclusion tasks, so that the chance performance was 24 responses. The single-sample *t*-test was used to determine which of the indexes was greater than 24.

In the SOA = 850 ms group, the extent of learning was greater than chance, *t*(1,22) = 12.30, *p* = 0.000 < 0.001, Cohen’s *d* = 2.56, which suggests that implicit sequence learning did occur. The correct inclusion-task response was not greater than chance, *p* = 0.075 > 0.05, but the incorrect exclusion-task response was greater than chance, *t*(1,22) = 3.25, *p* = 0.004 < 0.01, Cohen’s *d* = 0.68, which indicates that there were no correct inclusion-task response, but there were incorrect exclusion-task response. However, the classical PDP states that the correct inclusion-task response contains the incorrect exclusion-task response (Jacoby, 1991); that is, the correct inclusion-task response should be greater than or equal to the incorrect exclusion-task response. Thus, the classical PDP was inconsistent with the outcomes.

In the SOA = 1,350 ms group, the extent of learning was greater than chance, *t*(1,22) = 7.17, *p* = 0.000 < 0.001, Cohen’s *d* = 1.49, which suggests that implicit sequence learning occurred. The correct inclusion-task response was greater than chance, *t*(1,22) = 4.10, *p* = 0.000 < 0.001, cohen’ *d* = 0.85, but the incorrect exclusion-task response was not greater than chance, *p* = 0.479 > 0.05, which indicates that there were only correct inclusion-task response, but no incorrect exclusion-task response.

Further, the correct inclusion-task response and the incorrect exclusion-task response were not associated in the SOA = 850 ms group, *p* = 0.148 > 0.05, but were negatively associated in the SOA = 1,350 ms group, *r*(21) = −0.56, *p* = 0.005 < 0.01. Putting the two groups together, the two responses were negatively associated, *r*(44) = −0.46, *p* = 0.001. The classical PDP states that the correct inclusion-task response (*C* + *A*) contains the incorrect exclusion-task response (*A*); that is, they can be either positively (most likely), negatively or not associated, which depends on the relationship between *C* and *A*. Although the classical PDP could explain the lack of correlation and negative correlation results, the results could not validate the classical PDP. It was seen that the MET was better suited for the results, which states that the correct inclusion-task response (*C* + *A*_1_) and the incorrect exclusion-task response (*A*_2_) are mutually exclusive; that is, they can only be either negatively associated (most likely) or not correlated (because of random error), but they can never be positively associated.

#### Resting-State Brain Activity Related to the Classical PDP

Although both the correct inclusion-task response in the SOA = 850 ms group and the incorrect exclusion-task response in the SOA = 1,350 ms group were at chance, there might exist individual differences. Therefore, each SOA group was split half into high and low subgroups using the two responses as the criterion: this included high and low correct inclusion-task response subgroups and high and low incorrect exclusion-task response subgroups. The high and low subgroups differed significantly (independent-groups *t*-test, *p*s < 0.01), whereby the high subgroup performed greater than chance (*p*s < 0.001). This indicates that individual differences were present for the two responses, and it was meaningful to carry out Pearson correlation analyses between the resting-state brain activity and the two responses in each SOA group. The subgroups were only made to test the individual differences. Therefore, they were not further analyzed.

Pearson correlation analyses were made between the resting-state brain activity and the two responses in each SOA group (see Table 2). It is clear that in each group, brain areas related to the correct inclusion-task response and the incorrect exclusion-task response were either completely different for most brain areas or opposite for some brain areas, which indicates that the two responses were independent or competitive. These results were inconsistent with the classical PDP, which can be inferred that brain areas related to the two responses must, in part, overlap with each other, but were perfectly consistent with the MET, which states that the two responses are mutually exclusive with each other. Therefore, we analyzed resting-state brain activity related to the improved PDP in accordance with the MET.

**TABLE 2:** Correlations between resting-state ALFFs and the classical PDP.

SOA = 850 ms (*n* = 23)				SOA = 1,350 ms (*n* = 23)			
AAL brain area	ALFFs	*r*_in_	*r*_ex_	AAL brain area	ALFFs	*r*_in_	*r*_ex_
Frontal_Inf_Orb_L	0.81 ± 0.09	0.43*		Precentral_R	0.94 ± 0.08	−0.45*	
Olfactory_L	1.01 ± 0.06	0.56**		Frontal_Mid_Orb_L	0.64 ± 0.12	0.47*	
Rectus_L	0.94 ± 0.08	0.42*		Frontal_Inf_Tri_L	0.84 ± 0.08	0.48*	−0.44*
Insula_L	0.96 ± 0.04		−0.51*	Frontal_Inf_Orb_L	0.81 ± 0.09	0.58**	
Occipital_Sup_R	0.88 ± 0.06		−0.42*	Rolandic_Oper_L	0.88 ± 0.06	0.41*	
Fusiform_R	0.91 ± 0.05		−0.48*	Rolandic_Oper_R	0.93 ± 0.06		0.42*
Postcentral_L	0.85 ± 0.05	0.51*		Insula_L	0.96 ± 0.04	0.73**	
Angular_L	1.02 ± 0.06		0.43*	Insula_R	0.97 ± 0.05	0.46*	
Heschl_L	1.09 ± 0.13		−0.44*	Cingulum_Ant_R	0.94 ± 0.06	0.52*	
Cerebelum_Crus1_L	0.91 ± 0.16		−0.49*	Cingulum_Mid_L	0.96 ± 0.04	0.48*	
Cerebelum_Crus1_R	0.93 ± 0.14		−0.47*	Cingulum_Mid_R	0.96 ± 0.04	0.43*	
Cerebelum_4_5_R	1.11 ± 0.1		−0.43*	ParaHippocampal_L	1.13 ± 0.09	0.48*	−0.55**
Cerebelum_6_L	0.94 ± 0.07		−0.53*	Amygdala_L	1.02 ± 0.11	0.42*	
Cerebelum_6_R	0.91 ± 0.09		−0.69**	Fusiform_L	0.91 ± 0.04	0.44*	
Vermis_6	0.89 ± 0.08		−0.64**	Postcentral_L	0.85 ± 0.05	−0.48*	
Vermis_7	0.8 ± 0.08		−0.56**	Paracentral_Lobule_L	0.98 ± 0.1	−0.52*	0.43*
				Paracentral_Lobule_R	1.15 ± 0.23	−0.44*	
				Pallidum_L	0.81 ± 0.05		0.49*
				Pallidum_R	0.8 ± 0.05		0.45*
				Heschl_L	1.09 ± 0.13	0.43*	
				Temporal_Sup_L	1.04 ± 0.06		−0.43*
				Temporal_Pole_Sup_R	1.05 ± 0.12		−0.45*
				Temporal_Pole_Mid_L	0.78 ± 0.14		−0.43*
				Temporal_Pole_Mid_R	0.66 ± 0.1		−0.47*
				Vermis_7	0.8 ± 0.08		0.44*

r_in_ is the correlation between resting-state ALFFs and the correct inclusion-task response; r_ex_ is the correlation between resting-state ALFFs and the incorrect exclusion-task response. *p < 0.05, **p < 0.01. The p-values of correlations are in the Appendix.

### The Establishment of the Improved PDP in Accordance With the MET

#### The Improved PDP Analysis for Generation Tasks

Because the classical PDP did not conform with our results, we improved the logic and reanalyzed the data with the MET. There were 12 kinds of triplets in the SOC, each of which was repeated 6 times in the inclusion/exclusion tasks in the current study. Further, each of them had a given number of the correct inclusion-task response and the incorrect exclusion-task response for a participant, and chance performance for the inclusion/exclusion tasks would be one-third, namely 2 times. Using the MET to expand the classical PDP, we compared the relationship between the two responses for each triplet and defined four knowledge types (see Table 3).

**TABLE 3:** Four knowledge types of the improved PDP.

Types of knowledge	The correct inclusion-task response	The incorrect exclusion-task response	SOA = 850 ms (*M* ± *SD*, *n* = 23)	SOA = 1,350 ms (*M* ± *SD*, *n* = 23)
Non-acquisition of knowledge	≤2	≤2	2.83 ± 1.90	2.87 ± 1.71
Uncontrollable knowledge	≤2	≥3	3.87 ± 1.60	2.96 ± 2.03
Half-controllable knowledge	≥3	≥3	1.65 ± 1.56	1.74 ± 1.66
Controllable knowledge	≥3	≤2	3.65 ± 2.40	4.43 ± 2.81

(1) If both the two responses for a given kind of triplet were at chance (i.e., less than or equal to 2), this kind of triplet was defined as non-acquisition of any knowledge. Figuratively speaking, it is like a striking donkey who does not work, whether it is needed or not.
(2) If the correct inclusion-task response for a given kind of triplet was at chance, but the incorrect exclusion-task response was greater than chance (i.e., greater than or equal to 3), this kind of triplet was defined as uncontrollable knowledge; that is, participants could not use the knowledge to choose rule-based positions in the inclusion task, which required participants to choose rule positions, but the knowledge made participants automatically choose rule positions in the exclusion task, which required participants to avoid choosing rule positions. It is like a naughty donkey, who does not work when it is needed, but does work when it is not needed. Uncontrollable knowledge is inconsistent with the classical PDP.
(3) If both the two responses for a given kind of triplet were greater than chance, this kind of triplet was defined as half-controllable knowledge. It is like an overly enthusiastic donkey, who always works, whether it is needed or not. Half-controllable knowledge is consistent with the classical PDP.
(4) If the correct inclusion-task response for a given kind of triplet was greater than chance, but the incorrect exclusion-task response was at chance, this kind of triplet was defined as controllable knowledge. It is like a well-trained donkey, who works when it is needed, and does not work when it is not needed.

The participants’ control of the four knowledge types (degree of consciousness) was gradually improved.

For the SOA = 850 ms group, a repeated-measures analysis of variance was carried out for the four types of knowledge. Sphericity was non-significant (*p* = 0.165 > 0.05), and there was a significant main effect of knowledge type, *F*(3,20) = 4.84, *p* = 0.004 < 0.01, $\eta_{\text{p}}^{2}$ = 0.180. After Bonferroni correction for multiple comparisons, there was significantly less half-controllable knowledge than uncontrollable knowledge (*p* = 0.003 < 0.01) and controllable knowledge (*p* = 0.038 < 0.05). A similar analysis for the SOA = 1350 ms group (sphericity: *p* = 0.080 > 0.05) revealed a significant main effect of knowledge type, *F*(3,20) = 4.76, *p* = 0.005 < 0.01, $\eta_{\text{p}}^{2}$ = 0.178. After Bonferroni correction, there was significantly less half-controllable knowledge than controllable knowledge (*p* = 0.019 < 0.05). There was no difference for the four knowledge types between the two groups by one-way ANOVA (*p*s = 0.935, 0.098, 0.855, 0.316 > 0.05).

#### Resting-State Brain Activity Related to the Improved PDP

**FIGURE 2:**
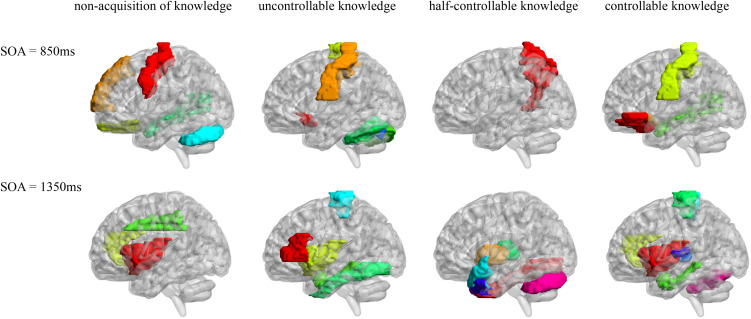
AAL brain areas related to the four knowledge types of the improved PDP. The brain areas were visualized with the BrainNet Viewer (http://www.nitrc.org/projects/bnv/) (Xia et al., 2013).

Pearson correlation analyses were made between the resting-state brain activity and the four knowledge types of the improved PDP in each SOA group (see Table 4 and Figure 2).

**TABLE 4:** Correlations between resting-state ALFFs and the four knowledge types of the improved PDP.

SOA = 850 ms (*n* = 23)						SOA = 1,350 ms (*n* = 23)					
AAL brain area	ALFFs	*r*_non_	*r*_un_	*r*_half_	*r*_con_	AAL brain area	ALFFs	*r*_non_	*r*_un_	*r*_half_	*r*_con_
Frontal_Inf_Oper_L	0.84 ± 0.06	−0.42*				Frontal_Inf_Tri_L	0.87 ± 0.04		−0.52*		
Frontal_Inf_Orb_L	0.81 ± 0.09				0.54**	Rolandic_Oper_R	0.88 ± 0.03		0.51*		
Olfactory_L	1.01 ± 0.06		−0.44*		0.49*	Insula_L	0.92 ± 0.05	−0.47*	−0.45*		0.47*
Frontal_Sup_Medial_R	1.01 ± 0.07	0.46*				Insula_R	0.99 ± 0.05	−0.49*			0.43*
Rectus_L	0.94 ± 0.08	−0.53**				Cingulum_Ant_R	0.9 ± 0.05	−0.48*			0.43*
Rectus_R	0.86 ± 0.07	−0.42*				Cingulum_Mid_L	0.96 ± 0.05	−0.48*			
Postcentral_L	0.85 ± 0.05		−0.48*		0.55**	ParaHippocampal_L	1.12 ± 0.11		−0.51*		0.51*
Precuneus_L	1.09 ± 0.05			0.42*		Fusiform_L	0.87 ± 0.03		−0.45*		
Paracentral_Lobule_L	0.98 ± 0.1		−0.47*			Fusiform_R	0.86 ± 0.03			0.45*	
Temporal_Mid_R	0.94 ± 0.04	−0.54**			0.46*	Paracentral_Lobule_L	1.01 ± 0.16		0.53**		−0.46*
Cerebelum_Crus1_L	0.91 ± 0.16	0.47*				Paracentral_Lobule_R	1.11 ± 0.22				−0.42*
Cerebelum_Crus1_R	0.93 ± 0.14		−0.53**			Putamen_L	0.78 ± 0.04			0.46*	
Cerebelum_6_L	0.94 ± 0.07		−0.43*			Pallidum_L	0.83 ± 0.04			0.47*	
Cerebelum_6_R	0.91 ± 0.09		−0.52*			Pallidum_R	0.81 ± 0.05			0.44*	
Vermis_7	0.8 ± 0.08		−0.43*			Thalamus_L	1.02 ± 0.1			0.45*	−0.50*
						Heschl_L	1 ± 0.06				0.48*
						Heschl_R	1.09 ± 0.07				0.44*
						Temporal_Pole_Sup_L	1.11 ± 0.08			−0.44*	
						Temporal_Pole_Sup_R	1.01 ± 0.08			−0.43*	
						Temporal_Pole_Mid_L	0.73 ± 0.09			−0.43*	
						Temporal_Pole_Mid_R	0.58 ± 0.04			−0.45*	
						Cerebelum_Crus1_L	0.89 ± 0.11			0.44*	
						Cerebelum_6_R	0.92 ± 0.06				−0.45*
						Vermis_7	0.8 ± 0.05			0.46*	

r_non_ is the correlation between resting-state ALFFs and non-acquisition of knowledge; r_un_ is the correlation between resting-state ALFFs and uncontrollable knowledge; r_half_ is the correlation between resting-state ALFFs and half-controllable knowledge; r_con_ is the correlation between resting-state ALFFs and controllable knowledge. *p < 0.05, **p < 0.01. The p-values of correlations are in the Appendix.

In the SOA = 850 ms group, it was seen that: first, some frontal lobe (Frontal_Sup_Medial_R) and cerebellum (Cerebelum_Crus1_L) were positively related to non-acquisition of knowledge. Some frontal lobes (Frontal_Inf_Oper_L and Rectus_L and R) and temporal lobe (Temporal_Mid_R) were negatively related to non-acquisition of knowledge. Second, there was no brain area positively related to uncontrollable knowledge. Some frontal lobes (Olfactory_L and Paracentral_Lobule_L), parietal lobe (Postcentral_L), cerebellum (Cerebelum_Crus1_R and Cerebelum_6_L and R) and vermis (Vermis_7) were negatively related to uncontrollable knowledge. Third, the left precuneus (Precuneus_L) was positively related to half-controllable knowledge. There was no brain area negatively related to half-controllable knowledge. Fourth, some frontal lobe (Frontal_Inf_Orb_L and Olfactory_L), parietal lobe (Postcentral_L) and temporal lobe (Temporal_Mid_R) were positively related to controllable knowledge. There was no brain area negatively related to controllable knowledge. Fifth, most relevant brain area for the four knowledge types were different, except that the right middle temporal gyrus was negatively related to non-acquisition of knowledge, but was positively related to controllable knowledge; and that left olfactory and left postcentral gyrus were negatively related to uncontrollable knowledge, but was positively related to controllable knowledge.

In the SOA = 1,350 ms group, the following observations were made: first, there was no brain area positively related to non-acquisition of knowledge. Some insulas (Insula_L and R) and cingulate gyrus (Cingulum_Ant_R and Cingulum_Mid_L) were negatively related to no-acquisition. Second, some frontal lobes (Rolandic_Oper_R and Paracentral_Lobule_L) were positively related to uncontrollable knowledge. Some frontal lobe (Frontal_Inf_Tri_L), insula (Insula_L), hippocampal (ParaHippocampal_L) and fusiform (Fusiform_L) were negatively related to uncontrollable knowledge. Third, some fusiform (Fusiform_R), putamen (Putamen_L), pallidum (Pallidum_L, R), thalamus (Thalamus_L), cerebellum (Cerebelum_Crus1_L), and vermis (Vermis_7) were positively related to half-controllable knowledge. Some temporal lobes (Temporal_Pole_Sup_L and R and Temporal_Pole_Mid_L and R) were negatively related to half-controllable knowledge. Fourth, some insulas (Insula_L and R), cingulate gyrus (Cingulum_Ant_R), hippocampal (ParaHippocampal_L), and temporal lobes (Heschl_L and R) were positively related to controllable knowledge. Some frontal lobes (Paracentral_Lobule_L and R), thalamus (Thalamus_L), and cerebellum (Cerebelum_6_R) were negatively related to controllable knowledge. Fifth, most relevant brain areas for the four knowledge types were different, except that the left insula was negatively related to non-acquisition of knowledge and uncontrollable knowledge, but was positively related to controllable knowledge; the right insula and right anterior cingulate cortex were negatively related to non-acquisition of knowledge, but were positively related to controllable knowledge; the left parahippocampal was negatively related to uncontrollable knowledge, but was positively related to controllable knowledge, however, left paracentral lobule was on the contrary; and the left thalamus was positively related to half-controllable knowledge, but was negatively related to controllable knowledge.

As SOA increased, it was seen that: first, the brain areas related to non-acquisition of knowledge changed from frontal lobes, temporal lobe and cerebellum to insulas and cingulate gyrus. There were no common relevant brain areas. Second, the brain areas related to uncontrollable knowledge changed from some frontal lobes, parietal lobe, cerebellum and vermis to other frontal lobe, parietal lobe, insula, hippocampal and fusiform. There was only one common relevant brain area–the left paracentral lobule, but its correlation coefficient ranged from negative to positive. Third, the brain areas related to half-controllable knowledge changed from precuneus to some fusiform, putamen, pallidum, thalamus, temporal lobes, cerebellum and vermis. There were no common relevant brain areas. Fourth, the brain areas related to controllable knowledge changed from some frontal lobe, parietal lobe, and temporal lobe to other frontal lobe, some insulas, cingulate gyrus, hippocampal, thalamus, temporal lobe, and cerebellum. There were no common relevant brain areas.

## Discussion

### The Test for the Classical PDP and the Establishment of the Mutually Exclusive Theory

The classical PDP states that the correct inclusion-task response contains the incorrect exclusion-task response (Jacoby, 1991); that is, the correct inclusion-task response should be greater than, or equal to, the incorrect exclusion-task response, and they can be positively (most likely) associated, negatively associated or not associated, which depends on the relationship between *C* and *A*. The present study found that in the SOA = 850 ms group, the correct inclusion-task response was at chance, but the incorrect exclusion-task response occurred greater than chance, which was inconsistent with the classical PDP, but was perfectly consistent with the MET. In the SOA = 850 ms group, the two responses were not correlated; however, in the SOA = 1350 ms group, and in the two groups together, the two responses were in contrast to each other, which could be explained by the classical PDP, but could not prove the classical PDP. The MET was better suited to explain the results, which state that the correct inclusion-task response (*C* + *A*_1_) is mutually exclusive with the incorrect exclusion-task response (*A*_2_); that is, they can only be either negatively associated (most likely) or not associated (because of random error), but cannot be positively associated. As SOA increased, the correct inclusion-task response changed from at chance to above chance, but the incorrect exclusion-task response changed from above chance to at chance. This suggests that higher SOAs increased the level of consciousness, and that the representation theory quality by Cleeremans and Jiménez (2002) was applicable to SOA.

ALFFs in the resting-state could predict the correct inclusion-task response and the incorrect exclusion-task response of the classical PDP in the two groups. But in each group, brain areas related to the two responses were either completely different (for most brain areas) or opposite (for some brain areas), which indicates that they were independent or competitive. These neuroimaging results were also inconsistent with the classical PDP, which suggested that brain areas related to the two responses must, in part, overlap. However, the MET was more suited to explain the neuroimaging results. Some frontal lobes, parietal lobes, fusiform gyrus, temporal lobes include the hippocampus, cingulate gyrus, insula, basal ganglia and cerebellum in the resting-state were related to the correct inclusion-task response or the incorrect exclusion-task response, many of which were consistent with the results of the task-state researches (Destrebecqz et al., 2005; Guo et al., 2008; Wilkinson et al., 2009; Penhune and Steele, 2012; Yang and Li, 2012; Hardwick et al., 2013; Huang et al., 2017). It proved that the rationale of the resting-state was applicable to the present study of implicit sequence learning consciousness.

In summary, the present study, for the first time tested the classical PDP by behavior and neuroimaging data, and found that the results were inconsistent with the model. However, the results were perfectly consistent with the MET proposed by the present study. Of course, the MET need to be tested under more conditions, including task-state fMRI, more RSI (SOA), more or less blocks, novel stimuli, among others.

### The Establishment of the Improved PDP and Its Component Analysis by the Mutually Exclusive Theory

For the first time, an improved PDP was created to categorize the 12 kinds of triplets in the SOC as delineating four knowledge types, namely non-acquisition of knowledge, uncontrollable knowledge, half-controllable knowledge, and controllable knowledge. The participants’ control of the four kinds of knowledge (degree of consciousness) was gradually improved. Only half-controllable knowledge was in accordance with implicit knowledge as defined by the classical PDP, but it was present to an equal or lesser extent than uncontrollable knowledge, which is contrary to the classical PDP, but is consistent with the MET. It is clear that either correct inclusion response or incorrect exclusion response was a mixture of four knowledge types.

According to the MET, each of the four knowledge types is equal to its correct inclusion response (*C* + *A*_1_) plus its incorrect exclusion response (*A*_2_), which is equal to (*C* + *A*_1_) + *A*_2_. *A*_2_ is automatic implicit knowledge against the exclusion task requirement, and *A*_1_ is controlled implicit knowledge meeting the inclusion task requirement.

There were four levels. The first level was as follows:

Non-acquisition of knowledge = (*C* + *A*_1_) + *A*_2_ = 0 + 0 = 0, because both *C* + *A*_1_ and *A*_2_ = 0.
Uncontrollable knowledge = (*C* + *A*_1_) + *A*_2_ = 0 + *A*_2_ = *A*_2_, because *C* + *A*_1_ = 0, but *A*_2_ ≠ 0.
Half-controllable knowledge = (*C* + *A*_1_) + *A*_2_ = *C* + *A*_1_ + *A*_2_, because both *C* + *A*_1_ and *A*_2_ ≠ 0.
Controllable knowledge = (*C* + *A*_1_) + *A*_2_ = (*C* + *A*_1_) + 0 = *C* + *A*_1_, because *C* + *A*_1_ ≠ 0, but *A*_2_ = 0.

If we consider *C* and *A*_1_, the second level was as follows:

Half-controllable knowledge contained three cases: first, half-controllable knowledge is equal to *A*_1_ + *A*_2_, when *C* = 0, but *A*_1_ ≠ 0; second, half-controllable knowledge is equal to *C* + *A*_1_ + *A*_2_, when both *C* and *A*_1_ ≠ 0; third, half-controllable knowledge is equal to *C* + *A*_2_, when *C* ≠ 0, but *A*_1_ = 0.
Controllable knowledge also contained three cases: first, controllable knowledge is equal to *A*_1_, when *C* = 0, but *A*_1_ ≠ 0; second, controllable knowledge is equal to *C* + *A*_1_, when both *C* and *A*_1_ ≠ 0; third, controllable knowledge is equal to *C*, when *C* ≠ 0, but *A*_1_ = 0.

The two levels mentioned above assume that neither *C* nor *A*_1_ was less than 0. If we assume that both *C* and *A*_1_ are less than 0, the third level was seen to consist of the a state where the non-acquisition of knowledge might have some knowledge, when *C* ≥ 0 (right controlled knowledge) and *A*_1_ ≤ 0 (wrong automatic knowledge), or vice versa. There was a confrontation between *C* and *A*_1_ in the inclusion task. Similarly, there might be more complicated cases for the other three knowledge types.

However, all the three levels did not consider the sub-components of *C*, *A*_1_, or *A*_2_. Because the participants could get the right and wrong knowledge which had opposite effects on the responses, the forth level was as follows: each of the four knowledge types were equal to (*C*_r_ − *C*_w_ + *A*_1r_ − *A*_1w_) + (*A*_2r_ − *A*_2w_), in which *C*_r_, *A*_1r_ and *A*_2r_ were the right sub-components of *C*, *A*_1_ and *A*_2_, and *C*_w_, *A*_1w_, and *A*_2w_ were the wrong sub-components of *C*, *A*_1_, and *A*_2_. The fourth level is the most complete and detailed, but it needs to be further explored and validated.

In summary, the improved PDP used by the present study could help acquire more precise knowledge than the classical PDP, and the four knowledge types could be explained perfectly by the MET. There was no behavioral difference of each knowledge type between two SOA groups.

### The Related Brain Areas of the Improved PDP and Their Interpretation by the Mutually Exclusive Theory

The improved PDP can facilitate the acquisition more rigorous and precise knowledge, especially in the study of brain mechanisms. The ALFFs in the resting-state could predict the four knowledge types of the improved PDP in two groups.

In the SOA = 850 ms group, the following was noted: first, some areas of the consciousness network (some frontal lobe, Destrebecqz et al., 2005; Sami et al., 2014) and some areas of the motor network (some cerebellum, Penhune and Steele, 2012; Hardwick et al., 2013) were positively related to non-acquisition of knowledge, which means that they were unfavorable for the acquisition of knowledge. It is possible that the irrelevant and wrong consciousness and self-consciousness by frontal lobe and the motor fixation by cerebelum (fixation on key-press and did not get the rules between key-presses) hindered knowledge acquisition. Some areas of the consciousness network (some frontal lobes and temporal lobe) were negatively related to non-acquisition of knowledge, which means they were helpful for knowledge acquisition (Destrebecqz et al., 2005; Squire, 2009). Second, there was no brain area positively related to uncontrollable knowledge. Some areas of the consciousness network (some frontal lobes, parietal lobe) and motor network (some cerebelum and vermis) were negatively related to uncontrollable knowledge (Destrebecqz et al., 2005; Huang et al., 2017; Sami et al., 2014). Third, some areas of the consciousness network (left precuneus, Dehaene et al., 2014) were positively related to half-controllable knowledge. There was no brain area negatively related to half-controllable knowledge. Fourth, some areas of the consciousness network (some frontal lobe, parietal lobe, and temporal lobe) were positively related to controllable knowledge. There was no brain area negatively related to controllable knowledge. Fifth, most brain areas related to the four knowledge types were different, which means that the four knowledge types were mostly independent in terms of the brain areas related to their particular function. The right middle temporal gyrus (consciousness area) was helpful for both knowledge acquisition and controllable knowledge. Uncontrollable knowledge and controllable knowledge were competitive in Left olfactory and left postcentral gyrus (consciousness areas).

In the SOA = 1,350 ms group, it was found that: first, there was no brain area positively related to non-acquisition of knowledge. Some areas of the consciousness network (some insulas and cingulate gyrus, Destrebecqz et al., 2005; Yang and Li, 2012) were negatively related to non-acquisition of knowledge, which means the insulas and cingulate gyrus facilitated knowledge acquisition. Second, some areas of the somatic sensorimotor network (right rolandic operculum and left paracentral lobule) were positively related to uncontrollable knowledge. Some areas of consciousness network (some frontal lobe, insula, hippocampal, and fusiform, Destrebecqz et al., 2005; Squire, 2009; Yang and Li, 2012; Huang et al., 2017) were negatively related to uncontrollable knowledge. Third, some areas of the consciousness network (Some fusiform and thalamus, Schröter et al., 2012; Huang et al., 2017), some areas of the implicit learning network (putamen and pallidum, Penhune and Steele, 2012; Sami et al., 2014) and some areas of the motor network (cerebelum and vermis) were positively related to half-controllable knowledge. Some temporal lobes responsible for consciousness were negatively related to half-controllable knowledge. Further, half-controllable knowledge contained complex components. Fourth, some areas of the consciousness network (some insulas, cingulate gyrus, hippocampal, and temporal lobe) were positively related to controllable knowledge. Some areas of the consciousness network (some frontal lobe and thalamus, maybe the irrelevant and wrong consciousness and self-consciousness) and cerebellum (responsible for motor process) were negatively related to controllable knowledge. Fifth, brain areas associated with the four knowledge types were different, which means that the four knowledge types were mostly independent in terms of the brain areas related to their particular function. The left insula responsible for consciousness was helpful for both knowledge acquisition and controllable knowledge, but was adverse for uncontrollable knowledge. Right insula and right anterior cingulate cortex were helpful for both knowledge acquisition and controllable knowledge. Uncontrollable knowledge and controllable knowledge were competitive in left parahippocampal and left paracentral lobule. Half-controllable knowledge and controllable knowledge were competitive in the left thalamus.

The participants’ control of the four knowledge types (degrees of consciousness) gradually improved. Correspondingly, the brain areas in the resting-state positively associated with the four knowledge types gradually changed from the sensory and motor network to the somatic sensorimotor network (right rolandic operculum and left paracentral lobule), and then to the implicit learning network (precuneus, fusiform, thalamus, putamen, and pallidum), and then to the consciousness network (the frontal lobes, parietal lobes, temporal lobes, cingulate gyrus, and insula); the brain areas in the resting-state negatively associated with the four knowledge types gradually changed from the consciousness network to the sensory and motor network. If the sensory and motor network of the participants was strong, but the consciousness network was weak, they could press keys quickly and smoothly without needing to acquire sequence knowledge. In the case that the somatic sensorimotor network of the participants was strong, but the sensory and motor network and the consciousness network were weak, the participants should be hijacked by somatic sensorimotor feelings only to acquire the uncontrollable knowledge. In the case that the implicit learning network were strong, and the conscious network were weak, half-controllable knowledge could be acquired. In the case that the consciousness network was strong, controllable knowledge could be acquired. The relationship between individual differences in brain areas and the four knowledge types was revealed, which indicates that there were different consciousness ability types for different participants because of their different dominant brain areas (Woolhouse and Bayne, 2000; Zhang et al., 2014). It would be useful to further explore this with a large sample in the future.

According to the MET, each of the four knowledge types is equivalent to (*C* + *A*_1_) + *A*_2_ = (*C*_r_ − *C*_w_ + *A*_1r_ − *A*_1w_) + (*A*_2r_ − *A*_2w_). Therefore, the brain areas associated with uncontrollable knowledge were the brain areas related to *A*_2_; the brain areas associated with half-controllable knowledge were the brain areas relevant to (*C* + *A*_1_) + *A*_2_; the brain areas associated with controllable knowledge were the brain areas related to (*C* + *A*_1_); and the brain areas associate with non-acquisition of knowledge (knowledge acquisition) were the brain areas related to the total of the other three knowledge types. It should be noted that each of the *C*, *A*_1_, and *A*_2_ of the four knowledge types was not the same one, because they belonged to different triplets. There are three hypotheses for the relationship of the cognitive components of the four knowledge types:

(1) The homogeneous hypothesis. The MET holds that each knowledge type can be separated into three components (*C*, *A*_1_, and *A*_2_), but it is not determined whether the components of the four knowledge types are homogeneous. If they are homogeneous, we can use the brain areas associated with each of the four knowledge types to analyze the others. For example, the brain areas positively associated with uncontrollable knowledge (*A*_2_) were the somatic sensorimotor areas; so, we can speculate that the somatic sensorimotor areas were also the brain areas positively associated with the *A*_2_ component of half-controllable knowledge. The brain areas positively associated with controllable knowledge (*C* + *A*_1_) were consciousness areas; so, we can speculate that consciousness areas were also the brain areas positively associated with the (*C* + *A*_1_) component of half-controllable knowledge.
(2) The inhomogeneous hypothesis. However, it was found that the brain areas associated with the four knowledge types were independent or competitive with each other, indicating that the same components among the four knowledge types might not be homogeneous. For example, the *A*_2_ component of uncontrollable knowledge and the *A*_2_ component of half-controllable knowledge were not homogeneous, among other such instances. Therefore, we cannot use the brain areas associated with one of the four types of knowledge to analyze another. Instead, we should further develop new measurement methods to isolate the three components of each knowledge type and then examine brain areas associated with them.
(3) The indivisible hypothesis. The two hypotheses above are based on the premise that each knowledge type can be split into three components, according to the MET. The three components can be further split into sub-components. However, there is a hypothesis states that they cannot be separated; instead, they are complete and indivisible, which was also realized with the neuroimaging results. If it is true, the MET should be improved. Fortunately, the incorrect exclusion-task response behaviorally separated *A*_2_ component of each knowledge type, suggesting that it was at least partly separable for each knowledge type, and the formulae of the MET were partially validated. Some new behavior measurement methods need to be developed to divide the correct inclusion-task response into *C* and *A*_1_ of each knowledge type in order to fully validate the MET. Of course, there is another hypothesis to be explored, which states that each knowledge type only can be split into *A*_2_ and (*C* + *A*_1_), but that (*C* + *A*_1_) cannot be split into *C* and *A*_1_.

As SOA increased, the brain areas associated with almost all the four knowledge types changed, which means that SOA changed the brain areas associated with each knowledge type (Cleeremans and Jiménez, 2002; Destrebecqz et al., 2005; Huang et al., 2017), and that the same knowledge types might not be homogeneous among brain mechanisms between the two SOA groups, although they had similar behavioral characteristics. Those results can be well interpreted by the MET to argue that there are many cases for a knowledge type (see the 2–4 levels in section “The Establishment of the Improved PDP and Its Component Analysis by the Mutually Exclusive Theory”). There was one common brain area associated with uncontrollable knowledge: the left paracentral lobule; however, its correlation coefficient was ranged from negative to positive. In contrast, there was no difference in behavioral data of the four knowledge types between two groups, which indicates that behavioral data were not sensitive as neuroimaging data.

In conclusion, the inhomogeneous hypothesis of the MET, which stated that the components of the four knowledge types were not homogeneous, and that knowledge types were not homogeneous between the two SOA groups either, is best suited to interpret behavioral and neuroimaging data. We can see that the four knowledge types, their three components and their behavioral and brain mechanisms were incredibly complicated and need to be further investigated.

A comparison of the classical PDP and the MET was made in this study, with many indexes. It is clear that MET is the better theory (see Table 5). In the future, the MET can also be applied to explore consciousness of implicit cognition, such as consciousness of perception, implicit memory, implicit artificial grammar learning, implicit social cognition, and so on.

**TABLE 5:** A comparison of the classical PDP and the MET.

	The classical PDP	The MET	Relationship to the results
Logic and operations	The correct inclusion-task response contain the incorrect exclusion-task response.	Mutually exclusive	MET (consistent)
Implicit knowledge types	Only one implicit knowledge type A	Many implicit knowledge types *A*_1_, *A*_2_	MET (consistent)
Quantity of the two responses	The correct inclusion-task response should be greater than or equal to the incorrect exclusion-task response.	Unrestricted	MET (consistent)
Correlation of the two responses	Unrestricted	Only negatively correlated or uncorrelated	MET (better suitability)
Brain areas of the two responses	Must in part overlap	Independent or competitive	MET (consistent)
Accuracy and diversity of knowledge	General C and A for the total of the 12 triplets	Four knowledge types for each triplet	MET (better suitability)
Interpretation of behavioral and neuroimaging results of the improved PDP	None	Each knowledge type = (*C* + *A*_1_) + *A*_2_ = (*C*_r_ − *C*_w_ + *A*_1r_ − *A*_1w_) + (*A*_2r_ − *A*_2w_). Inhomogeneous hypothesis	MET (consistent)

## Conclusion

The present study used behavioral and resting-state neuroimaging data to test and improve the classical PDP to the mutually exclusive theory (MET) in implicit sequence learning.

(1) Behavioral data and neuroimaging data demonstrated that the classical PDP has not been validated. In the SOA = 850 ms group, the correct inclusion-task response was at chance, but the incorrect exclusion-task response occurred greater than chance. In the SOA = 850 ms group, the two responses were not correlated, but in the SOA = 1,350 ms group and putting the two groups together, the two responses were in contrast to each other. In each group, brain areas whose amplitude of low frequency fluctuations (ALFFs) in the resting-state related to the two responses were either completely different or opposite to one another. However, the results were perfectly consistent with the MET proposed by the present study which suggests that the correct inclusion-task response is equal to the correct exclusion-task response is equal to *C* + *A*_1_, and the incorrect exclusion-task response is equal to *A*_2_. *C* denotes the controlled response and *A*_1_ and *A*_2_ denote two different automatic responses.
(2) The improved PDP was proposed to categorize the 12 kinds of triplets in the SOC as delineating four knowledge types, namely non-acquisition of knowledge, uncontrollable knowledge, half-controllable knowledge, and controllable knowledge with the MET. ALFFs in the resting-state could predict the four knowledge types of the improved PDP among two groups. The participants’ control of the four knowledge types (degree of consciousness) gradually improved. Correspondingly, the brain areas in the resting-state positively related to the four knowledge types, gradually changed from the sensory and motor network to the somatic sensorimotor network, and then to the implicit learning network, and then to the consciousness network. The brain areas in the resting-state negatively related to the four knowledge types gradually changed from the consciousness network to the sensory and motor network. As SOA increased, the brain areas associated with almost all the four knowledge types changed.
(3) The inhomogeneous hypothesis of the MET is best suited to interpret behavioral and neuroimaging data; it states that the same components among the four knowledge types are not homogeneous, and the same knowledge types are not homogeneous between the two SOA groups.

## Data Availability Statement

The datasets generated for this study will not be made publicly available. The datasets for this manuscript are not publicly available because there are still too many valuable results to be presented in this manuscript, and the authors will use the datasets for other analysis to write new manuscript. Requests to access the datasets should be directed to JZ, blade_kensin@163.com.

## Ethics Statement

The studies involving human participants were reviewed and approved by the Scientific Review Committee of Faculty of Psychology, Southwest University, China. The patients/participants provided their written informed consent to participate in this study.

## Author Contributions

JZ provided research ideas and financial support, and was responsible for research design, data collection and analysis, and article writing. XW was responsible for guiding fMRI research design, data collection and analysis and article writing, and provided brain imaging theoretical and technical support. JH was responsible for document collection, participant recruitment, and helped data collection. AC was responsible for guiding fMRI research design, data collection and analysis and article writing, supervising and reviewing the whole research process, and provide fMRI using. DL was responsible for guiding the design, implementation, data analysis and article writing of the whole research, and provided financial support. All authors contributed to the article and approved the submitted version.

## Conflict of Interest

The authors declare that the research was conducted in the absence of any commercial or financial relationships that could be construed as a potential conflict of interest.

## Appendix

**APPENDIX TABLE 1:** The *p*-values of correlations between resting-state ALFFs and the classical PDP.

SOA = 850 ms (*n* = 23)				SOA = 1,350 ms (*n* = 23)			
AAL brain area	ALFFs	*p*_in_	*p*_ex_	AAL brain area	ALFFs	*p*_in_	*p*_ex_
Frontal_Inf_Orb_L	0.81 ± 0.09	0.040		Precentral_R	0.94 ± 0.08	0.030	
Olfactory_L	1.01 ± 0.06	0.005		Frontal_Mid_Orb_L	0.64 ± 0.12	0.024	
Rectus_L	0.94 ± 0.08	0.044		Frontal_Inf_Tri_L	0.84 ± 0.08	0.020	0.038
Insula_L	0.96 ± 0.04		0.014	Frontal_Inf_Orb_L	0.81 ± 0.09	0.004	
Occipital_Sup_R	0.88 ± 0.06		0.049	Rolandic_Oper_L	0.88 ± 0.06	0.050	
Fusiform_R	0.91 ± 0.05		0.022	Rolandic_Oper_R	0.93 ± 0.06		0.045
Postcentral_L	0.85 ± 0.05	0.013		Insula_L	0.96 ± 0.04	0.000	
Angular_L	1.02 ± 0.06		0.039	Insula_R	0.97 ± 0.05	0.026	
Heschl_L	1.09 ± 0.13		0.038	Cingulum_Ant_R	0.94 ± 0.06	0.010	
Cerebelum_Crus1_L	0.91 ± 0.16		0.019	Cingulum_Mid_L	0.96 ± 0.04	0.020	
Cerebelum_Crus1_R	0.93 ± 0.14		0.023	Cingulum_Mid_R	0.96 ± 0.04	0.041	
Cerebelum_4_5_R	1.11 ± 0.1		0.040	ParaHippocampal_L	1.13 ± 0.09	0.019	0.007
Cerebelum_6_L	0.94 ± 0.07		0.010	Amygdala_L	1.02 ± 0.11	0.047	
Cerebelum_6_R	0.91 ± 0.09		0.000	Fusiform_L	0.91 ± 0.04	0.037	
Vermis_6	0.89 ± 0.08		0.001	Postcentral_L	0.85 ± 0.05	0.020	
Vermis_7	0.8 ± 0.08		0.006	Paracentral_Lobule_L	0.98 ± 0.1	0.010	0.039
				Paracentral_Lobule_R	1.15 ± 0.23	0.038	
				Pallidum_L	0.81 ± 0.05		0.017
				Pallidum_R	0.8 ± 0.05		0.030
				Heschl_L	1.09 ± 0.13	0.039	
				Temporal_Sup_L	1.04 ± 0.06		0.043
				Temporal_Pole_Sup_R	1.05 ± 0.12		0.032
				Temporal_Pole_Mid_L	0.78 ± 0.14		0.043
				Temporal_Pole_Mid_R	0.66 ± 0.1		0.025
				Vermis_7	0.8 ± 0.08		0.037

*p_in_ is the p-value of correlation between resting-state ALFFs and the correct inclusion-task response; p_ex_ is the p-value of correlation between resting-state ALFFs and the incorrect exclusion-task response.*

**APPENDIX TABLE 2:** The *p*-values of correlations between resting-state ALFFs and the four knowledge types of the improved PDP.

SOA = 850 ms (*n* = 23)						SOA = 1,350 ms (*n* = 23)					
AAL brain area	ALFFs	*p*_non_	*p*_un_	*p*_half_	*p*_con_	AAL brain area	ALFFs	*p*_non_	*p*_un_	*p*_half_	*p*_con_
Frontal_Inf_Oper_L	0.84 ± 0.06	0.045				Frontal_Inf_Tri_L	0.87 ± 0.04		0.011		
Frontal_Inf_Orb_L	0.81 ± 0.09				0.008	Rolandic_Oper_R	0.88 ± 0.03		0.012		
Olfactory_L	1.01 ± 0.06		0.037		0.018	Insula_L	0.92 ± 0.05	0.025	0.032		0.024
Frontal_Sup_Medial_R	1.01 ± 0.07	0.026				Insula_R	0.99 ± 0.05	0.017			0.042
Rectus_L	0.94 ± 0.08	0.010				Cingulum_Ant_R	0.9 ± 0.05	0.022			0.043
Rectus_R	0.86 ± 0.07	0.045				Cingulum_Mid_L	0.96 ± 0.05	0.020			
Postcentral_L	0.85 ± 0.05		0.019		0.006	ParaHippocampal_L	1.12 ± 0.11		0.014		0.013
Precuneus_L	1.09 ± 0.05			0.045		Fusiform_L	0.87 ± 0.03		0.031		
Paracentral_Lobule_L	0.98 ± 0.1		0.025			Fusiform_R	0.86 ± 0.03			0.032	
Temporal_Mid_R	0.94 ± 0.04	0.008			0.028	Paracentral_Lobule_L	1.01 ± 0.16		0.009		0.028
Cerebelum_Crus1_L	0.91 ± 0.16	0.024				Paracentral_Lobule_R	1.11 ± 0.22				0.048
Cerebelum_Crus1_R	0.93 ± 0.14		0.009			Putamen_L	0.78 ± 0.04			0.026	
Cerebelum_6_L	0.94 ± 0.07		0.043			Pallidum_L	0.83 ± 0.04			0.025	
Cerebelum_6_R	0.91 ± 0.09		0.010			Pallidum_R	0.81 ± 0.05			0.034	
Vermis_7	0.8 ± 0.08		0.042			Thalamus_L	1.02 ± 0.1			0.031	0.016
						Heschl_L	1 ± 0.06				0.021
						Heschl_R	1.09 ± 0.07				0.036
						Temporal_Pole_Sup_L	1.11 ± 0.08			0.034	
						Temporal_Pole_Sup_R	1.01 ± 0.08			0.038	
						Temporal_Pole_Mid_L	0.73 ± 0.09			0.040	
						Temporal_Pole_Mid_R	0.58 ± 0.04			0.030	
						Cerebelum_Crus1_L	0.89 ± 0.11			0.036	
						Cerebelum_6_R	0.92 ± 0.06				0.033
						Vermis_7	0.8 ± 0.05			0.027	

*p_non_ is the p-value of correlation between resting-state ALFFs and non-acquisition; p_un_ is the p-value of correlation between resting-state ALFFs and uncontrollable knowledge; p_half_ is the p-value of correlation between resting-state ALFFs and half-controllable knowledge; p_con_ is the p-value of correlation between resting-state ALFFs and controllable knowledge.*
